# Human Hendra Virus Encephalitis Associated with Equine Outbreak, Australia, 2008

**DOI:** 10.3201/eid1602.090552

**Published:** 2010-02

**Authors:** Elliott G. Playford, Brad McCall, Greg Smith, Vicki Slinko, George Allen, Ina Smith, Frederick Moore, Carmel Taylor, Yu-Hsin Kung, Hume Field

**Affiliations:** Pathology Queensland, Brisbane, Queensland, Australia (E.G. Playford); University of Queensland, Brisbane (E.G. Playford, B. McCall); Forensic and Scientific Services, Brisbane (G. Smith, I. Smith, F. Moore, C. Taylor); Brisbane South Public Health, Brisbane (B. McCall, V. Slinko); Princess Alexandra Hospital, Brisbane (E.G. Playford, G. Allen); Department of Primary Industries and Fisheries, Brisbane (Y.-H. Kung, H. Field)

**Keywords:** Hendra virus, Henipavirus, Paramyxoviridae, ribavirin, Australia, encephalitis, equines, viruses, research

## Abstract

A recent Hendra virus outbreak at a veterinary clinic in Brisbane, Queensland, Australia, involved 5 equine and 2 human infections. In contrast to previous outbreaks, infected horses had predominantly encephalitic, rather than respiratory, signs. After an incubation period of 9–16 days, influenza-like illnesses developed in the 2 persons before progressing to encephalitis; 1 died. Both patients were given ribavirin. Basal serum and cerebrospinal fluid levels were 10–13 mg/L after intravenous administration and 6 mg/L after oral administration (isolate 90% inhibitory concentration 64 mg/L). Both patients were exposed to infected horses, 1 during the late incubation period in a horse. The attack rate for veterinary clinic staff exposed to infected horses was 10%. An isolate from this outbreak showed genetic heterogeneity with isolates from a concurrent, but geographically remote, outbreak and from previous outbreaks. Emergence of Hendra virus is a serious medical, veterinary, and public health challenge.

## Abstract 2

Emergence of this virus is a serious medical, veterinary, and public health challenge.

The genus *Henipavirus* of the family *Paramyxoviridae* contains 2 recently described viruses, Hendra virus and Nipah virus, whose natural reservoir is fruit bats of the genus *Pteropus* (*1*). Hendra virus has caused serious respiratory infections in horses and respiratory and neurologic infections in humans. After 2 Hendra virus outbreaks in 1994, which involved 22 horses and 3 humans (*2*,*3*), a total of 7 additional equine infections and 1 human infection were documented up to 2008 (*4*,*5*); all occurred in coastal Queensland and northern New South Wales in Australia. Of the 4 persons with documented Hendra virus infection, 2 recovered from influenza-like illnesses (ILIs) (*2*,*5*) and 2 died, 1 from respiratory failure (*2*) and 1 from encephalitis 13 months after initial aseptic meningitis (*3*). The estimated incubation period in humans of 7–8 days was based on these cases.

## The Outbreak

In early July 2008, a veterinary practice (clinic) in Thornlands, Queensland, was quarantined after 2 acutely ill horses were provisionally diagnosed with Hendra virus infection. Four horses eventually died from the infection, and another was humanely killed after it recovered, in accordance with established national veterinary practice (*6*). In contrast to previous equine outbreaks, horses in this outbreak showed predominantly neurologic (ataxia, head tilt, limb paresis), rather than respiratory, symptoms (*1*). We report 2 additional human cases of Hendra virus encephalitis, 1 fatal, in veterinary workers associated with this equine outbreak.

### Patient 1

A 33-year-old man (equine veterinarian) at the clinic had a 2-day history of an ILI (fever, myalgia, and headache) in mid-July 2008. Clinical examination showed only a fever (38°C); mild neutropenia (0.7 × 10^9^ cells/L) and thrombocytopenia (79 × 10^9^ cells/L); a normal chest radiograph; and negative PCR results for respiratory viruses, including influenza, on a nasopharyngeal aspirate (NPA) specimen. Hendra virus RNA was detected by reverse transcription–PCR (RT-PCR) in serum and NPA specimens. The patient remained clinically well and showed defervescence on day 4 of illness.

By day 5, mild confusion, ataxia, bilateral ptosis, but no other neurologic signs, developed. Magnetic resonance imaging (MRI) showed multifocal (bilateral, cerebral, cortical, right pontine, scattered white matter) hyperintense lesions on T2 flair images associated with evidence of infarction on diffusion weighted images. A cerebrospinal fluid (CSF) sample had a leukocyte count of 4 × 10^6^ cells/L, a protein level of 600 mg/L, and a glucose level of 3.7 mmol/L; Hendra virus RNA was detected by RT-PCR. An electroencephalogram (EEG) showed bilateral high-voltage slow waves but no epileptiform activity. He was treated with intravenous ribavirin (30 mg/kg initial dose, then 15 mg/kg every 6 h for 4 days, and 8 mg/kg every 8 h thereafter); enteric aspirin (100 mg/day); and because of progressive confusion and ataxia, intravenous dexamethasone (4 mg every 6 h).

**Figure 1:**
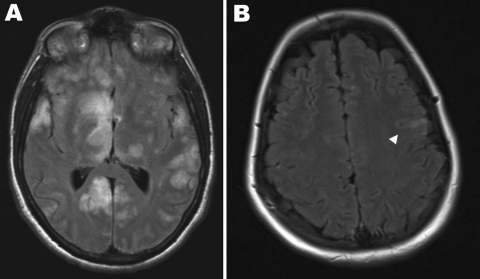
Magnetic resonance imaging scans of the brains of 2 patients with Hendra virus encephalitis, Australia, 2008. A) Patient 1 on day 18 of illness, showing cortical and subcortical hyperintense foci. B) Patient 2 on day 25 of illness, showing hyperintense foci in the left precentral gyrus (arrowhead).

Over the next 4 days (to day 10) progressive neurologic signs and high-grade fever developed, culminating in a generalized partial tonic-clonic seizure. During the next 4 weeks, the patient required mechanical ventilation, remained febrile, and despite control of seizure activity and minimal sedation, remained deeply unconscious with minimally reactive pupils. Treatment with ribavirin was stopped after 12 days of therapy when the patient’s hemoglobin level decreased to 76 g/L. Serial MRI showed marked progression with innumerable cortical, subcortical, and brainstem hyperintense foci on T2 flair images with matching diffusion restriction, and leptomeningeal enhancement (Figure 1, panel A). Serial EEGs showed an absence of a stable background rhythm, slow wave activity with periodic sharp discharges, and a right-sided epileptogenic focus. CSF on day 28 had a leukocyte count of 84 × 10^6^ cells/L, a protein level of 650 mg/L, and a glucose level of 4.3 mmol/L; Hendra virus RNA was not detected by RT-PCR. He died on day 40 of illness. A postmortem examination was not performed because a risk assessment conducted by Queensland Health concluded that an examination could not be performed in Queensland.

### Patient 2

A 21-year-old woman (veterinary nurse) at the same clinic was observed 4 days after patient 1 with a 3-day history of an ILI. Results of clinical examination were unremarkable apart from a fever (39°C). Results of initial investigations, including complete blood examination, chest radiography, and EEG, were normal. Hendra virus RNA was detected in serum and NPA specimens. She was treated with intravenous ribavirin (30 mg/kg initial dose, then 15 mg/kg every 6 h) and aspirin (100 mg/day). On day 12 of illness, 96 hours after defervescence, encephalitic manifestations (mild confusion, dysarthria, ataxia, and bilateral ptosis) developed. Results of MRI and a CSF specimen were normal; Hendra virus was not detected by RT-PCR.

Over the next 12 days, signs of encephalitis deteriorated (ataxia, dysarthria) before stabilizing. Serial MRI showed scattered, small, hyperintense, cortical and subcortical foci on T2 flair images and leptomeningeal enhancement (Figure 1, panel B), and EEGs showed severe diffuse encephalopathy with high-amplitude slow waves. Clinical and EEG improvement ensued, and the patient was discharged on day 37 with mild residual ataxia. By then, she had received 32 days of intravenous ribavirin and had steady-state basal serum ribavirin levels of 10.1 mg/L and 13.1 mg/L and a CSF level of 9.9 mg/L (all obtained while she was receiving 15 mg/kg every 6 h; J. Ray, pers. comm.).

Treatment with ribavirin was continued at a dosage of 600 mg orally every 8 h (basal level 6.2 mg/L; J. Ray, pers. comm.), and she remained clinically well during 20 weeks of follow-up. Repeat MRI (at days 69 and 135 and month 11) showed marked resolution in signal abnormalities, and EEGs (at days 69 and 182) also showed considerable improvement. A CSF sample obtained at month 12 showed normal results, including a negative result for Hendra virus by RT-PCR.

## Results

### Virologic and Serologic Findings

Serial quantitative RT-PCR for Hendra virus RNA (*7*) in serum, NPA, and urine specimens and serial immunofluorescent antibody assay and microsphere immunoassay for immunoglobulin (Ig) G and IgM against Hendra virus (*8*) in serum were performed for both patients (Appendix Table). Seroconversion of IgG against Hendra virus occurred on days 5 and 11 in patients 1 and 2 respectively, and Hendra virus RNA was detected up to days 14 and 23, respectively.

**Figure 2:**
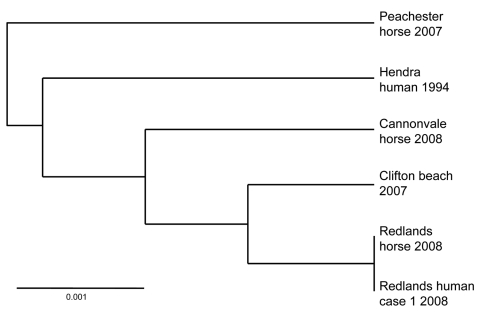
Phylogram showing relationships between Hendra virus isolates, Australia, 2008, based on medium gene sequence. Scale bar indicates nucleotide substitutions per site.

Direct PCR sequencing of clinical material from both patients was used to obtain nucleotide sequences for the nucleoprotein, matrix, glycoprotein, and accessory protein genes and the intragenic junctions. Results showed that Hendra virus associated with this outbreak was distinct from Hendra virus in a concurrent equine outbreak in Cannonvale in northern Queensland, Australia, and from viruses in previous outbreaks (Figure 2).

Virus isolated from Vero E6 cells (CRL-1586; American Type Culture Collection, Manassas, VA, USA) cultured with an NPA sample obtained on day 5 of illness of patient 2 was used to test in vitro susceptibility to ribavirin (*9*). The 90% inhibitory concentration of ribavirin for this isolate was 64 mg/L.

### Human Public Health Investigations and Responses

After confirmation of equine Hendra virus cases, 83 contacts of infected horses (37 veterinary clinic staff, 25 owners/handlers, and 21 Department of Primary Industries staff) and 8 contacts of human case-patients (6 domestic contacts, and 2 healthcare workers) were contacted for assessment and counseling. Nonspecific respiratory or gastrointestinal symptoms developed in 16 contacts of infected horses; none had Hendra virus RNA or antibodies against this virus in convalescent-phase serum samples (obtained >2 weeks after last exposure to infected horses or humans). One veterinary worker had percutaneous blood exposure while performing euthanasia on the Hendra virus–infected horse that recovered. This person was treated with a 5-day course of intravenous ribavirin (30 mg/kg initial dose, then 15 mg/kg every 6 h) within 4 h after exposure. Serologic follow-up up to 25 weeks showed no evidence of infection with Hendra virus.

Degree of exposure to infected horses and use of personal protective equipment (PPE) were assessed in 28 veterinary clinic staff. Twenty (including the 2 patients) reported contact with potentially infected equine body fluids; the Hendra virus attack rate for exposed staff was 10%. Fourteen identified high-risk exposures (potential exposure through unprotected mucous membrane, broken skin, or respiratory route), among whom self-reported use of PPE (respiratory droplet and mucosal protection) was low (7%). The other 6 staff identified low-risk exposures (intact skin). High-risk exposures identified among the 2 infected patients included performing daily nasal cavity lavage during the last 3 days of the incubation period on 1 infected horse (9 and 11 days before illness onset in patients 1 and 2, respectively) and participating in a necropsy of another infected horse (patient 1) 16 days before illness onset.

## Discussion

Unlike previously recognized equine Hendra virus infections (*1*), this outbreak involved predominantly neurologic, rather than respiratory, equine symptoms. This finding may have contributed to delayed recognition of the etiologic cause among the sick horses and thus unprotected exposure of human contacts.

Of the 4 persons previously infected with Hendra virus, 1 died from multiorgan failure, another died from encephalitis 13 months after aseptic meningitis, and 2 others survived after ILIs. The 2 patients described in this report also had initial ILIs. However, soon after apparent clinical improvement, including defervescence, encephalitis developed in both patients. The MRI changes showed widespread cortical, subcortical, and deep white matter involvement, similar to those described in a previous Hendra virus encephalitis case (*3*) and Nipah virus encephalitis cases (*10*,*11*). These changes are consistent with the neuropathogenesis of *Henipavirus*-related encephalitis (widespread viral endothelial infection with resultant vasculitis, syncytial giant cell formation, and ischemic necrosis) (*12*). Although encephalitis developed in both patients 1–4 days after initial clinical improvement of the initial ILI and concurrent with the appearance of Hendra virus–specific antibodies, the ability of Henipaviruses to inhibit interferon signaling (*13*,*14*) is a major virulence factor that enables evasion of host responses. The other major phenomenon, described for 1 case of Hendra virus infection and in >5% and >10% of cases of Nipah virus encephalitic and nonencephalitic infections, respectively, is relapsed or late-onset encephalitic manifestations (*3*,*15*). However, the pathogenesis of this latency and understanding these clinical manifestations remain incomplete.

Given the previous demonstration of an in vitro effect of ribavirin against Hendra virus (*16*) and a reported survival benefit associated with its use (predominantly by the enteral route of administration) compared with historical controls in Nipah virus encephalitis (*17*), our patients received treatment with this drug. For these patients, the high-dose intravenous regimen was used, which was similar to that used for patients with Lassa fever (*18*). Although anemia developed in patient 1, the drug was well tolerated by patient 2. Furthermore, given a previously reported case of late-onset Hendra virus encephalitis (*3*), patient 2 was given prolonged oral therapy (to month 8), which was well tolerated. The in vivo efficacy of ribavirin against Hendra virus remains unknown, although this drug delayed but did not prevent deaths caused by Nipah virus in a hamster model (*19*).

Although basal serum and CSF ribavirin concentrations >10 mg/L (during administration of ribavirin, 15 mg/kg every 6 h intravenously) and basal serum ribavirin concentrations of 6 mg/L (during administration of 8 mg/kg every 8 h orally) were documented, these concentrations may be inadequate given the results of in vitro susceptibility testing. The 90% inhibitory concentration of ribavirin against one of the human virus isolates was 64 mg/L, which was similar to that reported for an original human isolate from 1994 (*20*).

Treatment with ribavirin was initiated early (before onset of encephalitis) for patient 2 but later (upon onset of encephalitis) in patient 1. However, the influence of this timing on outcome remains unclear, particularly given the uncertainty about activity of ribavirin against Hendra virus and other possible confounding factors. For example, patient 1 had different and possibly greater quantitative initial exposure to Hendra virus, earlier onset of encephalitis, and corticosteroid therapy that may have suppressed virus clearance. We also administered intravenous ribavirin prophylactically for 5 days in another person after a percutaneous blood exposure to a horse documented as positive for Hendra virus by RT-PCR. This regimen was well tolerated, and follow-up assessments have not demonstrated seroconversion.

We emphasize that the efficacy of ribavirin as therapy or prophylaxis remains, at best, uncertain. However, given the absence of other available therapies, and the tolerability of ribavirin, we advocate that it be given early in clinical illness and be considered where high-risk exposure to Hendra virus has occurred. However, because only 6 cases have been recognized in humans, the criteria for high-risk exposure remain undefined.

This absence of established therapies underscores the need for early recognition of equine Hendra virus infection, implementation of infection control precautions, and prevention of horse-to-human transmission. Although the attack rate for humans exposed to infected horses in this outbreak (10%) was similar to that in the other documented stable outbreak that caused human infection (at which time the causative pathogen had not been identified) (*2*), upon recognition of the outbreak and implementation of protective measures, no further horse-to-human transmission occurred.

Because this outbreak involved hitherto unrecognized equine manifestations with predominant neurologic disease in the relative absence of respiratory signs, equine case definitions have been revised to include either respiratory or neurologic manifestations or both (*21*). The degree of nucleotide sequence variation observed between Hendra virus isolates from this and previous outbreaks suggests greater genomic variation than previously assumed. Whether the prominent encephalitic manifestations observed in this outbreak reflect this genetic variability require further assessment.

Detailed exposure histories from our 2 patients suggest that the likely incubation period was 9–16 days, which was longer than previously estimated. Furthermore, both patients performed nasal cavity lavage of a horse during the 3 days before its onset of symptoms of infection with Hendra virus. This finding suggests that horses may be infectious late in their incubation period, as observed with other Paramyxoviridae (e.g., measles).

The modes of horse-to-human transmission of Hendra virus are not known completely, but likely result from direct contact with respiratory secretions and other equine tissue and fluids or from droplet or aerosol exposure. Furthermore, difficulties associated with clinical recognition of equine Hendra virus infection present challenging infection control implications for equine veterinary practice. Therefore, routine adoption of infection control precautions encompassing hand washing, use of gloves and other forms of PPE, and a high index of clinical suspicion for equine Hendra virus infection are required. Although experience with human Hendra virus infection is relatively limited, no evidence exists of human-to-human transmission. However, persistence of Hendra virus RNA in NPA samples throughout the first 2 weeks of illness indicates that healthcare workers potentially exposed to respiratory secretions should adopt PPE use to prevent exposure to droplets.

In conclusion, we describe severe Hendra virus infection in 2 veterinary workers, 1 who died, after exposure to infected horses. Given the absence of other established therapies, ribavirin was administered. However, further understanding of the pathogenesis of neurologic disease is required to guide potential therapies. These evolving equine clinical manifestations and the inevitable and increasing interactions between the natural host of the virus (fruit bat species) and horses will continue to challenge veterinary, public health, and medical communities.

## Supplementary Material

Appendix Table Virologic and serologic results for 2 patients with Hendra virus encephalitis, Australia, 2008*
