# Supplementary material for: Human Hendra Virus Encephalitis Associated with Equine Outbreak, Australia, 2008
Source: Emerg Infect Dis. 2010 Feb;16(2):219–23. doi: 10.3201/eid1602.090552 (PMC2957996; doi:10.3201/eid1602.090552)
Supplement: Appendix Table — Virologic and serologic results for 2 patients with Hendra virus encephalitis, Australia, 2008* [file 09-0552_appT-s1.pdf]

Appendix Table. Virologic and serologic results for 2 patients with Hendra virus encephalitis, Australia, 2008*

Day of illness	Patient 1								Patient 2							
	Clinical data†	RNA			MIA		IFA titer		Clinical data‡	RNA			MIA		IFA titer	
		Serum	Urine	NPA	IgM	IgG	IgM	IgG		Serum	Urine	NPA	IgM	IgG	IgM	IgG
2		+			–	–	<8	<8								
3		+		+	–	–	16	<8								
4	1			–						+		+	–	–	<8	<8
5	2	+	+	-	+	+	128	16	6	–	–	+	–	–	<8	<8
6	3	–	+	-	+	+	256	64		+	+	+	–	–	<8	<8
7		+	+	-	+	+	256	64		+	+	+	–	–	<8	<8
8		–		+	+	+	256	64	7	–	+	+	–	–	<8	<8
9	4	–		+	+	+	64	256		–	+	+	–	–	<8	<8
10		–	+	–	+	+	128	256		–	+	+	–	–	32	<8
11										+	–	+	+	–	32	16
12		–	+	–	+	+	128	256	8	–	+	–	+	+	32	32
13		–	+	–	+	+	128	256		+	–	–	+	+	32	16
14		–	+	–	+	+	128	256	9	+	–	+	+	+	32	16
15										–	–	+	+	+	32	16
16	5	–	–	–			256	256		–	–	–	+	+	32	32
17										+			+	+	64	32
18										+	–		+	+	64	64
19		–	–		+	+	256	256		–	–		+	+	128	64
23										–	+		+	+	128	64
25										–	–		+	+	128	64
31										–		–	+	+	128	256
35		–	–		+	+	1,024	256		–			+	+	256	256

*NPA, nasopharyngeal aspirate; MIA, microsphere immunoassay; Ig, immunoglobulin; IFA, immunofluorescent antibody.
A positive result for RNA indicates a sample that had a cycle threshold value <40 in a real-time PCR, and a positive result in the MIA indicates a sample that had a positive:negative ratio ≥45.
†1, defervescence; 2, onset of encephalitis and start of treatment with ribavirin; 3, start of treatment with dexamethasone; 4, fever recrudescence; 5, treatment with ribavirin was stopped.
‡6, start of treatment with ribavirin; 7, defervescence; 8, onset of encephalitis; 9, fever recrudescence.
